# A simplified explanation for the frameshift mutation that created a novel C-terminal motif in the *APETALA3 *gene lineage

**DOI:** 10.1186/1471-2148-6-30

**Published:** 2006-03-24

**Authors:** Elena M Kramer, Huei-Jiun Su, Cheng-Chiang Wu, Jer-Ming Hu

**Affiliations:** 1 Dept. of Organismic and Evolutionary Biology, Harvard University, Cambridge MA 02138, USA; 2 Institute of Ecology and Evolutionary Biology, National Taiwan University, Taipei, Taiwan

## Abstract

**Background:**

The evolution of type II MADS box genes has been extensively studied in angiosperms. One of the best-understood subfamilies is that of the *Arabidopsis *gene *APETALA3 *(*AP3*). Previous work has demonstrated that the ancestral paleo*AP3 *lineage was duplicated at some point within the basal eudicots to give rise to the paralogous *TM6 *and eu*AP3 *lineages. This event was followed in eu*AP3 *orthologs by the replacement of the C-terminal paleoAP3 motif with the derived euAP3 motif. It has been suggested that the new motif was created by an eight-nucleotide insertion that produced a translational frameshift.

**Results:**

The addition of 25 eudicot *AP3 *homologs to the existing dataset has allowed us to clarify the process by which the euAP3 motif evolved. Phylogenetic analysis indicates that the eu*AP3*/*TM6 *duplication maps very close to the base of the core eudicots, associated with the families Trochodendraceae and Buxaceae. We demonstrate that although the transformation of paleoAP3 into euAP3 was due to a frameshift mutation, this was the result of a single nucleotide deletion. The use of ancestral character state reconstructions has allowed us to demonstrate that the frameshift was accompanied by few other nucleotide changes. We further confirm that the sequence is evolving as coding region.

**Conclusion:**

This study demonstrates that the simplest of genetic changes can result in the remodeling of protein sequence to produce a kind of molecular 'hopeful monster.' Moreover, such a novel protein motif can become conserved almost immediately on the basis of what appears to be a rapidly generated new function. Given that the existing data on the function of such C-terminal motifs are somewhat disparate and contradictory, we have sought to synthesize previous findings within the context of the current analysis and thereby highlight specific hypotheses that require further investigation before the significance of the euAP3 frameshift event can be fully understood.

## Background

An increasing body of research has demonstrated that changes in gene regulation play a major role in the evolution of morphological form (reviewed [1-3]). That is not to say, however, that the evolution of coding sequence does not also contribute. Multiple examples from both plants and animals demonstrate that even minor changes in coding sequence can impact both biochemical and developmental functions (e.g., [4-7]). Interestingly, a common theme among many of these examples is gene duplication, which serves to release resultant paralogs from the selective pressures experienced by the single ancestral locus. In order to begin to understand the process by which non-synonymous mutation leads to changes in gene function, we need to be able to isolate such changes and characterize the pattern of sequence evolution in detail. This is facilitated by a thorough understanding of taxonomic and gene lineage evolution as well as a relatively recent evolutionary timescale. All of these criteria are met by the *APETALA3 *(*AP3*) lineage of type II MADS box genes.

Members of the type II MADS box family control many important aspects of plant development (reviewed [8]). Extensive phylogenetic analyses have identified multiple subfamilies, which are particularly well understood in the seed plants (reviewed [9]). This interest was largely triggered by the central role that type II MADS box genes play in the genetic program controlling floral organ identity. The so-called ABC model [10] describes how floral organ identity is determined by an overlapping set of three gene activities that produce distinct combinatorial codes: A class genes code for first whorl sepals; A+B, for second whorl petals; B+C, for third whorl stamens; and C alone, for fourth whorl carpels. Subsequent studies have identified additional critical gene classes, including the "E" class that acts in all floral whorls to facilitate the function of A, B and C class genes [11,12]. All but one of the ABCE class loci are type II MADS box genes [13], which are also known as MIKC MADS box genes due to the canonical structure displayed by the members. Starting at the N-terminal end of the gene, the 'M' or MADS domain is highly conserved across eukaryotes, and mediates DNA binding and protein dimerization [14,15]. The next two regions, referred to as I and K, are primarily involved with protein dimerization [14], while the last, the C domain, has been associated with a number of different functions. These include mediating higher-order interactions among MADS protein dimers [16,17], transcriptional activation [18,19], and post-translational modification [20]. A notable feature of the C-terminal domain is that although it shows a lower degree of overall sequence conservation than the other regions, each of the major MIKC subfamilies possesses short, highly conserved diagnostic motifs at their C-terminal end (reviewed [21,22]). In the majority of cases, the specific function of these motifs remains unknown.

As our understanding of the evolution of MIKC MADS box genes has grown, it has become increasingly clear that their evolutionary history is one of frequent gene duplication across all phylogenetic levels (reviewed [9,23]). One subfamily that demonstrates this phenomenon especially well is defined by the *APETALA3 *(*AP3*) and *PISTILLATA *(*PI*) gene lineages, which include the *Arabidopsis *petal and stamen identity genes of the same names. These two lineages are sister groups within the larger MIKC MADS gene family [24] and are the product of a gene duplication event that predated the diversification of the angiosperms [25-27]. Early studies recognized that there were, in fact, two paralogous lineages of *AP3*-like genes in the core eudicots: one termed eu*AP3 *that contains *AP3 *itself and the other named *TM6*, which lacks a representative in *Arabidopsis *but has been identified in many other core eudicot taxa [28,29]. Although clearly related, the eu*AP3 *and *TM6 *lineages have a number of distinct features, the most striking of which is their C-terminal motifs. In the *TM6 *and ancestral paleo*AP3 *lineages, the C-terminal motif has the consensus YGxHDLRLA (x indicating a variable site) [28]. This sequence, the paleoAP3 motif, is conserved throughout angiosperms and is recognizable in gymnosperm *AP3*/*PI *ancestors as well as the even more distantly related B_sister _lineage [30,31]. In the eu*AP3 *lineage, however, the paleoAP3 motif is completely absent and in its place is the so-called euAP3 motif with the consensus SDLTTFALLE [28]. The differences in this region and other sites reveal eu*AP3 *to be a divergent paralogous lineage relative to both its ancestral and sister lineages.

The patterns of sequence evolution associated with the eu*AP3*/*TM6 *duplication raise questions regarding the functional significance of the C-terminal motifs in general and the eu*AP3 *divergence in particular. From the biochemical standpoint, we can say with certainty that the euAP3 motif is important for proper *AP3 *function *in vivo*, and that the paleoAP3 and euAP3 motifs are not functionally equivalent [6,32]. In terms of the genes' developmental roles, the suggestion has been made that following the eu*AP3*/*TM6 *duplication, the eu*AP3 *lineage acquired a new role in petal development [6]. The evidence to support this conclusion is diverse, and includes: 1) the fact that the expression patterns of paleo*AP3 *orthologs in the petals of non-core eudicots are much more variable than those observed for eu*AP3 *representatives within the core eudicots [29,33]; 2) that a chimeric *AP3 *bearing a paleoAP3 motif is especially poor at promoting petal identity in *Arabidopsis *[6]; and 3) that the sole *TM6 *ortholog to be functionally characterized, *PhTM6 *from *Petunia*, only contributes to stamen identity ([34], Vandenbussche and Gerats, pers. comm). On the other hand, paleo*AP3 *orthologs are almost always expressed in petaloid organs (e.g., [35-37]) and appear to function in the identity of petal-derived organs in the grasses [38,39]. One explanation that could encompass all of the current evidence is to posit that although paleo*AP3 *members play variable roles in petal identity, this function was canalized at the base of the core eudicots in conjunction with changes in biochemical aspects of eu*AP3 *function and subsequent subfunctionalization in the *TM6 *lineage [40,41].

In regards to the evolution of the euAP3 motif itself, it was recently recognized that a frameshift event in the coding sequence of the paleoAP3 motif could generate components of the euAP3 motif [22]. The model of Vandenbussche et al. proposes that an eight nucleotide insertion contributed to the evolution of the euAP3 motif both by the addition of novel sequence and by causing a frameshift mutation. In the current study, we have sought to better establish the timing of the eu*AP3*/*TM6 *duplication event and the nature of the evolution of the euAP3 motif. The addition of 25 new *AP3 *homologs has particularly provided insight into the latter issue by demonstrating that the derivation of the euAP3 motif was even simpler than previously suggested. We conclude that a single nucleotide deletion transformed the ancestral paleoAP3 motif into the euAP3 motif with relatively few associated nucleotide changes. Furthermore, we provide evidence that the region is being conserved at the amino acid level, suggesting that the almost immediate conservation of the euAP3 motif was due to new function of the novel protein sequence.

## Results and discussion

### Characterization and phylogenetic analysis of *AP3 *homologs

**Figure 1:**
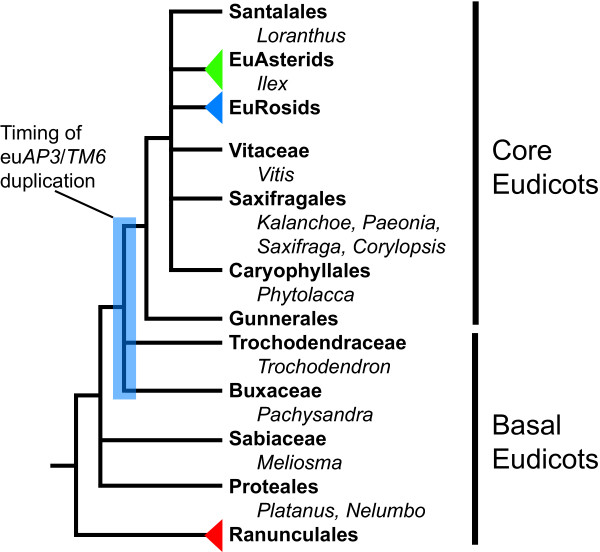
**Simplified eudicot phylogeny with newly sampled taxa. **Simplified eudicot phylogeny based on [42] and [43] with newly sampled taxa noted. The inferred timing of the eu*AP3*/*TM6 *duplication based on phylogenetic analyses of the current dataset (Fig. 2) is indicated by the blue box.

In an effort to better understand the evolution of the *AP3 *lineage in the eudicots, we used RT-PCR to isolate *AP3 *homologs from five taxa representing every lineage of the basal eudicots as well as eight taxa drawn from core eudicot lineages that had been poorly sampled (Fig. 1). This process yielded 25 *AP3 *homologs, 5 of which have been published in the context of previous studies [27,37] (see Additional file 1 for GenBank accession numbers). All of the basal eudicot loci exhibit well-conserved C-terminal paleoAP3 motifs (Additional file 2). *Pachysandra*, *Meliosma *and *Platanus *were found to express multiple paralogs with high degrees of sequence similarity, most likely indicating recent gene duplication events. As expected, two types of loci were identified in the core eudicots, some with paleoAP3 motifs and others with euAP3 motifs (Additional file 2). Both types were obtained from *Saxifraga*, *Corylopsis *and *Ilex*, but in the other five taxa we were only able to detect one of the two classes. Only paleoAP3-containing loci were found in *Phytolacca*, *Paeonia*, *Vitis *and *Loranthus*, while only euAP3-containing genes were identified in *Kalanchoe*. Multiple closely related paralogs were identified in *Kalanchoe*, *Phytolacca *and *Corylopsis*. The detection of only one *AP3 *class may have several different causes including actual paralog loss; low levels of paralog expression, which could hamper RT-PCR-based identification; and sequence divergence that prevented the success of current primer combinations.

**Figure 2:**
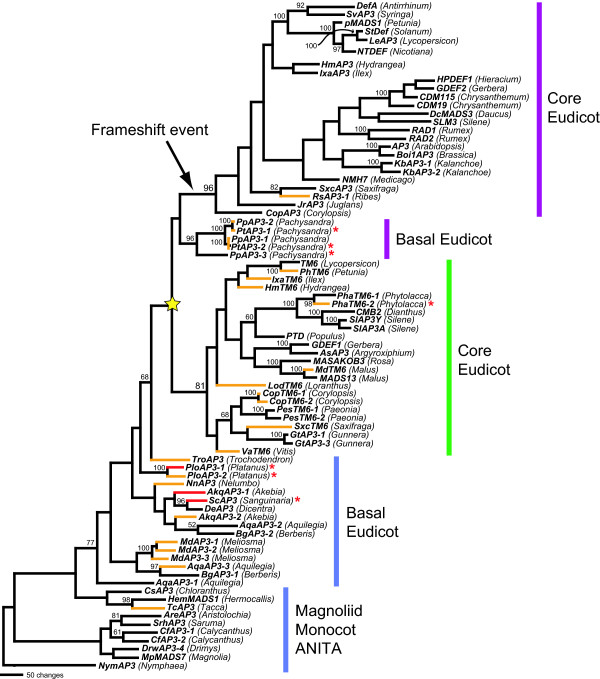
**Maximum likelihood phylogeny derived from analysis of the *AP3 *nucleotide dataset. **Bootstrap percentages (above 50) are placed at the nodes. The name of each taxon is in parentheses following the locus name. The node corresponding to the eu*AP3*/*TM6 *duplication is indicated with a star while the branch associated with the subsequent eu*AP3*-specific frameshift event is indicated with an arrow. Colored vertical bars on the right are used to indicate the paralog lineage membership of the adjacent loci: the purple bars represent the eu*AP3 *lineage; the green bar, the *TM6 *lineage; and the blue bars, the ancestral paleo*AP3 *lineage. The phylogenetic positions of the associated taxa are denoted as Core Eudicot, Basal Eudicot, Magnoliid, Monocot or ANITA (See Additional file 1, [94] and [95]). Colored branches are used to indicate the "frameshift potential" of each locus: black branches mean that a single nucleotide frameshift in the paleoAP3 or euAP3 motif would recover 0–3 amino acids of the other motif; orange branches, 4–6 amino acids; and red branches, seven or more amino acids. For instance, as shown in Fig. 3A, the first reading frame of *PloAP3-1 *encodes a perfect paleoAP3 motif but the second reading frame would produce a motif with seven out of the ten euAP3 motif residues, and, therefore, the *PloAP3-1 *branch is red. In contrast, the second reading frame of the *AreAP3 *paleoAP3 motif would have only two amino acids similar to the euAP3 motif, which is indicated by a black branch for *AreAP3*. Additionally, some paleo*AP3 *cDNAs would encode a positionally correct stop codon for a euAP3 motif in their second frame. These loci are denoted with red asterisks.

We performed phylogenetic analysis using maximum likelihood (ML) on a nucleotide dataset (Additional file 3) containing all of the new loci in addition to previously identified basal and core eudicot sequences, with magnoliid dicot, monocot and ANITA grade *AP3 *homologs serving as outgroups to the eudicot sequences (Fig. 2). The recovered phylogeny is consistent with previous analyses [26,28] in showing two major core eudicot lineages (eu*AP3 *and *TM6*) that were derived from an ancestral lineage (paleo*AP3*), which is represented in the basal eudicot and outgroup taxa. There is strong ML bootstrap support for the core eudicot eu*AP3 *and *TM6 *clades but little support for the other backbone nodes. Marginal support is seen for the clade containing *Trochodendron AP3*, the *Pachysandra AP3 *homologs and the other core eudicot sequences. The ML tree places *Trochodendron *and *Pachysandra *close to the gene duplication event that produced *euAP3 *and *TM6*. Based on a strict interpretation of the current phylogeny, this duplication would be inferred to have occurred after the divergence of Trochodendraceae but before the split of Buxaceae (star in Fig. 2). However, the lack of support for the backbone nodes allows alternative hypotheses. Most notably, the multiple loci from *Aquilegia *are not monophyletic (Fig. 2), suggesting additional duplications that may not be independent from eu*AP3 *and *TM6*. It has been demonstrated that there are at least three paralogous *AP3 *lineages in the Ranunculales [37] but this study did not test whether these events are related to that which gave rise to eu*AP3 *and *TM6*. Analysis of a dataset focused on complete sampling of the Ranunculales (including an additional 45 sequences) recovers all of the Ranunculid representatives as a single clade with moderate support (data not shown, Kramer, in prep). This indicates that the Ranunculid gene duplication events are, in fact, independent from that of eu*AP3*/*TM6*. While this increased sampling improves the resolution of the Ranunculid representatives, it is otherwise identical to the analysis shown in Fig. 2, both in terms of the positions of the Trochodendraceae and Buxaceae homologs, and in the lack of support for their positions.

The major departure of the current phylogeny from previous studies is the position of the *Pachysandra AP3 *homologs, representing sampling from two species, which are placed as sister to the eu*AP3 *lineage *s.s*. after the duplication event. This position is somewhat surprising given that none of the *Pachysandra *loci contain euAP3 motifs, which have previously been considered diagnostic for the eu*AP3 *lineage. However, in the I and K regions of the protein sequence (Additional file 3), the *Pachysandra AP3 *homologs share other character states that have been identified as eu*AP3 *lineage synapomorphies [28]. It should be noted that in maximum parsimony (MP) analyses, the *Pachysandra *loci sometimes are placed as an earlier branch, just before the eu*AP3*/*TM6 *duplication event (data not shown), underscoring the poorly supported position of these loci.

This analysis does allow us to make some conclusions regarding the timing of the eu*AP3*/*TM6 *duplication event. The duplication clearly occurred before the last common ancestor of all core eudicots, including the family Gunneraceae, which has been identified as sister to the traditionally defined core eudicot clade [42]. It seems likely that the duplication occurred after the early lineages of the basal eudicots, including the Ranunculales, Proteales and Sabiaceae. Based on the current analysis, we cannot determine with certainty how the timing of the duplication event related to the origin of the Trochodendraceae and Buxaceae lineages. Similarly, recent phylogenetic studies of the eudicots place these two families as sister to the core eudicots including Gunneraceae without strong support for their exact branching order (Fig. 1) [42,43]. Most likely, these difficulties reflect the very rapid diversification that occurred during this period of angiosperm evolution, which dates to ~95–115 mya [44].

### Evidence for a single nucleotide frameshift event at the base of the eu*AP3 *clade

**Figure 3:**
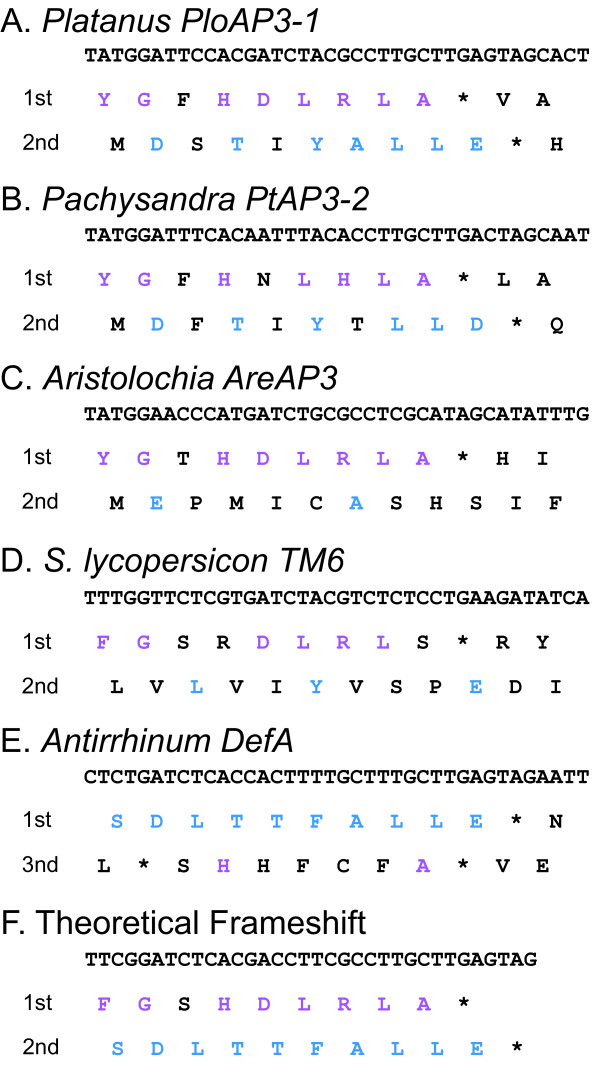
**Frameshift potentials of *Platanus PloAP3-1 *(A), *Pachysandra PtAP3-1 *(B), *Aristolochia AreAP3 *(C), *S. lycopersicon TM6 *(D), *Antirrhinum DefA *(E) and a theoretical paleoAP3-encoding sequence (F). **A-D, Nucleotide sequences of the paleoAP3-encoding regions of *Platanus PloAP3-1 *(A), *Pachysandra PtAP3-2 *(B), *Aristolochia AreAP3 *(C) and *S. lycopersicon TM6 *(D) with first and second predicted translation frames. E, Nucleotide sequence of the euAP3-encoding region of *Antirrhinum DefA *with first and third predicted translation frames. F, Nucleotide sequence of a theoretical DNA sequence that encodes a chemically conserved paleoAP3 motif in the first translational reading frame and a perfect euAP3 motif in the second translational reading frame. Chemical similarity with the paleoAP3 motif consensus YGxHDLRLA is indicated by purple letters while chemical similarity with the euAP3 motif consensus SDLTTFALLE is indicated by blue letters.

What is interesting about the current dataset is that all of the paleo*AP3 *lineage members and the *Pachysandra AP3 *homologs possess fairly normal paleoAP3 motifs with no clear sign of intermediates with the highly diverged euAP3 motif (Additional file 2). The explanation for this lack of 'missing links' has recently become apparent. In the course of characterizing the *AP3 *representatives from *Platanus *[37], we noticed that while the first reading frame encoded a perfect paleoAP3 motif, the second frame in the same region had the potential to encode an amino acid sequence with strong similarity to the euAP3 motif (Fig. 3A). The 3' UTR of *PloAP3-1 *even contains a stop codon in the correct frame and position. It has similarly been suggested by other researchers that a frameshift event transformed the paleoAP3 motif into the euAP3 motif, but this model posited an eight nucleotide insertion [22]. Examination of our basal eudicot sequences suggests a much simpler model whereby a single nucleotide deletion gave rise to the novel motif without the necessity for the insertion of new nucleotides. In fact, it is possible to construct a theoretical nucleotide sequence that encodes a chemically conserved paleoAP3 motif in the first reading frame and a perfect euAP3 motif in the second (Fig. 3F). We will subsequently refer to this phenomenon, the capacity of a given nucleotide sequence to simultaneously encode a paleoAP3 motif in the first reading frame and a recognizable euAP3 motif in the second, as 'frameshift potential.' Naturally occurring frameshift potential is particularly noticeable in other basal eudicot loci (Fig. 2, 3B). *AP3 *homologs from the magnoliid dicots, monocots or ANITA grade show little frameshift potential by comparison (Fig. 2, 3C). Similarly, core eudicot eu*AP3 *and *TM6 *lineage members exhibit relatively little frameshift potential (in the case of eu*AP3*, this would be a kind of 'reverse' frameshift potential to regenerate paleoAP3 sequence from euAP3; Fig. 2, 3D-E).

The phylogenetically-structured nature of euAP3/paleoAP3 frameshift potential suggests that it is dependent on patterns of codon usage and, therefore, that this region is behaving as normal coding region. This conclusion is significant since one possible explanation for the observed phenomenon is that the region is conserved at the nucleotide level rather than at the amino acid level, such as would be the case for something like a microRNA binding site, for example. The prediction of this scenario, however, is that the sequence should not evolve in a pattern typical of coding region, where the first and second codon positions exhibit lower nucleotide diversity than the third positions. An alternative model is that the region is subject to programmed translational frameshift, a phenomenon previously observed in fungal, prokaryotic, plastid and viral genomes (reviewed [45]). This process is associated with perturbations in the expected pattern of sequence evolution such that substitutions are concentrated in the third positions of the *original *reading frame rather than in the third positions of the new frame. In addition, the encoded amino acid sequence of the original frame is conserved (e.g., [46,47]). Thus, under the first hypothesis, the paleoAP3 sequence would be conserved at the nucleotide level and would not bear the hallmarks of coding sequence evolution, while under the second hypothesis, the sequence should evolve like coding sequence but in the original reading frame.

**Figure 4:**
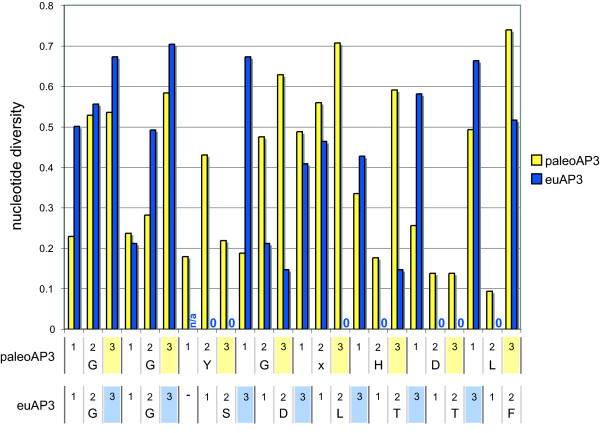
**Comparison of position-by-position nucleotide diversity values for paleoAP3 and euAP3 motif encoding loci (see also Add. Files 4-6). **The yellow bars indicate the values for a dataset including all *TM6 *lineage members and basal eudicot paleo*AP3 *loci. The codon positions of each nucleotide and the corresponding amino acids are shown immediately below the chart. Third positions are highlighted in yellow. The blue bars indicate the values for a dataset including all eu*AP3 *lineage members. The codon positions of each nucleotide and the corresponding amino acid are shown at the bottom, with third positions highlighted in blue. The position of the euAP3 frameshift is represented by a dash mark. Note that some of the euAP3 positions have zero nucleotide diversity. n/a = not applicable.

Our general observations, as well as those of others [22], are not consistent with these models but we wanted to test this further by directly analyzing patterns of nucleotide diversity in the region. Figure 4 shows a comparison of position-by-position nucleotide diversity values for the region spanning the inferred frameshift event (see also Additional file 6). In the codons before the frameshift, first and second positions generally show lower nucleotide diversity than third positions. This pattern is maintained in both the paleoAP3-encoding and frameshifted euAP3-encoding sequences. Comparison of the appropriate paleoAP3 and euAP3 positions reveals that when the first position nucleotides of the paleoAP3 motif become third positions in euAP3-encoding sequences, the nucleotide diversity generally increases. Similarly, third positions in the paleoAP3 motif show high diversity but these values tend to decrease when the nucleotide becomes shifted to the second position in the euAP3 motif. Overall, position-by-position nucleotide diversity differs between the paleoAP3 and euAP3 regions, which suggests that the patterns of conservation do change following the frameshift event. Taken together, these findings confirm that both regions show all of the evolutionary hallmarks of sequence that is being conserved at the amino acid level in the first reading frame, allowing us to reject both the nucleotide-level conservation and programmed translational frameshift hypotheses. We conclude, therefore, that the ancestral paleoAP3 motif, which was conserved over more than 200 million years [31], was completely replaced by a new amino acid motif via a single nucleotide deletion following gene duplication. The euAP3 motif appears to have been conserved due to its protein function rather than any underlying nucleotide-level function. This clarification of the model for euAP3 evolution has been facilitated by the greatly improved sampling of basal eudicot lineages, which, in turn, allowed the refinement of the *AP3 *alignment to include fewer indels than that used by Vandenbussche et al. [22].

**Figure 5:**
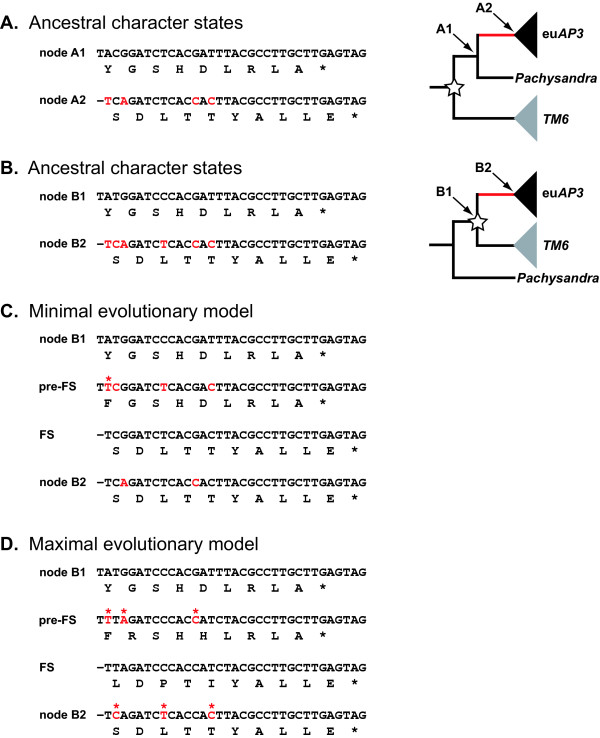
**Nucleotide ancestral character state reconstructions and evolutionary scenarios. **A, The MP ancestral character state reconstructions for the pre- and post-frameshift nodes (A1 and A2, respectively, as indicated to the right) of the recovered phylogeny (Fig. 2). These nucleotide sequences were recovered with the accelerated transitions (ACCTRAN) setting. Under the delayed transitions (DELTRAN) setting, the inferred sequences were identical to those shown in B. In the schematic phylogeny to the right, the star indicates the eu*AP3*/*TM6 *duplication node and the red branch denotes the timing of the frameshift event. B, The MP ancestral character state reconstructions for an alternative topology where the *Pachysandra *loci predate the eu*AP3*/*TM6 *duplication (again indicated by a star on the schematic phylogeny to the right). In this case, the frameshift occurred along the red branch immediately following the duplication event. Node B1 represents the duplication while B2 represents the ancestor of the eu*AP3 *clade. The sequences recovered with the ACCTRAN and DELTRAN settings were identical. C. Evolutionary scenario that minimizes the number of non-synonymous changes associated with the frameshift event. Each line represents stepwise changes that would have occurred during the transition from the sequence indicated as 'node B1' to that denoted 'node B2.' Under this model, four nucleotide changes occurred before the frameshift (line 'pre-FS') and two after (line 'node B2'), but only one of these changes was non-synonymous (indicated by red asterisk). D. Evolutionary scenario that maximizes the number of non-synonymous changes associated with the frameshift event. Again, each line represents stepwise changes that would have occurred during the transition from the sequence indicated as 'node B1' to that denoted 'node B2.' Under this model, three nucleotide changes occurred before the frameshift (line 'pre-FS') and three after (line 'node B2'), but all of these changes were non-synonymous (indicated by red asterisks). Scenarios in C and D are both based on the reconstructions shown in B. All nucleotide changes are indicated with red letters. FS = frameshift.

As shown in Fig. 3F, it is possible that the single nucleotide deletion was accompanied by few additional nucleotide changes. In an effort to investigate the potential range of nucleotide changes, we used MP and ML methods to reconstruct the ancestral nucleotide character states for critical nodes in the current *AP3 *phylogeny (Fig. 5). We also conducted the same analyses on alternative topologies to control for the fact that there is little or no support for the backbone of our phylogeny, (see Fig. 5 and Methods). Due to the high level of conservation in this region, the ancestral character state reconstructions were very similar for the MP and ML approaches, regardless of the models of substitution or the details of the topology. Based on these results, it appears that 4–6 nucleotide changes occurred coincidently with the frameshift event, which in the current phylogeny would be inferred to have occurred along the branch at the base of the eu*AP3 *clade after the separation of the Buxaceae (represented by *Pachysandra*; Fig. 2). We cannot predict the order of the nucleotide changes relative to the frameshift event, however; and due to the nature of the frameshift, some changes that are synonymous before the deletion event are non-synonymous after (and vice versa). Figs. 5C and 5D reconstruct two alternative scenarios using the ancestral character states shown in Fig. 5B (which infers six nucleotide changes). In Figs. 5C and 5D, each line represents a stepwise set of changes that could have occurred during the transition from the states reconstructed for node B1 to those recovered for node B2. The first scenario is a 'minimal' model in which only one of the six changes is nonsynonymous and this one change is chemically conservative (Fig. 5C). The second is a 'maximal' model where all six changes are nonsynonymous. In this case, three out of the nine paleoAP3 amino acids are changed before the frameshift and three of the ten euAP3 amino acids are changed afterward (a total of four of these changes are chemically non-conservative). Even under the 'maximal' model, the frameshift event was clearly more significant in terms of sequence remodeling, resulting in the replacement of all but one of the paleoAP3 amino acids. Overall, these findings demonstrate that it is possible for the euAP3 motif to have been generated by single nucleotide deletion without significant additional nonsynonymous changes.

### Evidence for independent frameshift events in the *AP3 *lineage

**Figure 6:**
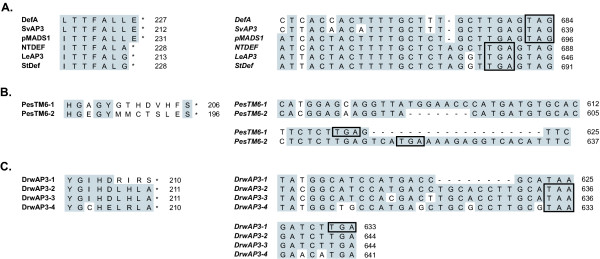
**Additional identified frameshift events in the *APETALA3 *lineage. **A, Amino acid (left) and corresponding nucleotide (right) alignments of the C-terminal regions of select Asterid eu*AP3 *cDNAs. Loci from *Antirrhinum *(*DefA*), *Syringa *(*SvAP3*) and *Petunia *(*pMADS1*) show the typical euAP3 motif but a single nucleotide insertion in the Solanaceous taxa *Nicotiana tobaccum *(*NTDEF*), *Solanum lycopersicon *(*LeAP3*) and *Solanum tuberosum *(*StDef*) has produced a one amino acid truncation. B, Amino acid (left) and corresponding nucleotide (right) alignments of the C-terminal regions of the *Paeonia suffructosa TM6 *lineage members *PesTM6-1 *and *PesTM6-2*. A seven nucleotide deletion in *PesTM6-2 *has given rise to a novel C-terminal motif that replaces the paleoAP3 motif (which is moderately conserved in *PesTM6-1*). There is an additional indel between the two loci in the region of the *PesTM6-2 *stop codon. C, Amino acid (left) and corresponding nucleotide (right) alignments of the C-terminal regions of the *Drimys winterii *paleo*AP3 *lineage members *DrwAP3-1*, *-2*, -*3 *and -*4*. An eight nucleotide deletion in *DrwAP3-1 *results in remodeling of the last four amino acids in the paleoAP3 motif. For A-C: Asterisks indicate translational stops. The stop codons used in the separate reading frames are boxed. Numbers at right indicate the position in the amino acid or nucleotide sequence of each locus. See also Additional Files 2 and 3.

The eu*AP3 *frameshift event seems so extraordinary that it naturally begs the question of how often this sort of thing happens. Similar events have been described in other MADS box genes lineages [22,48] as well as vertebrate gene families [49]. We examined the larger *AP3 *dataset for additional examples and found three (Fig. 6). The first we will consider is a single nucleotide insertion very close to the 3' end of the coding region in eu*AP3 *orthologs of the Solanaceae (Fig. 6A). Other eu*AP3 *loci from the Asterids, including the basal Solanaceous genus *Petunia *[50], show the complete euAP3 motif with a terminal glutamic acid. In comparison to these sequences, the eu*AP3 *homologs of more derived members of the Solanaceae have a single A insertion in the eighth codon of the euAP3 motif, which results in a single amino acid truncation of the motif. Such a minor change seems unlikely to have major biochemical significance, potentially explaining why the frameshifted form could be maintained. In contrast to this example, the other two instances are from taxa that have multiple recent *AP3 *paralogs. In *Paeonia*, there are two *TM6 *lineage members that share 91% identity at the nucleotide level. Their C-terminal regions are completely divergent, however, with *PesTM6-1 *having a recognizable paleoAP3 motif while *PesTM6-2 *has only the first tyrosine of the consensus (Fig. 6B). Examination of the nucleotide alignment reveals two indels in the 3' end of the coding region, the more significant of which is a 7-nucleotide deletion in *PesTM6-2 *that falls within the first codon of the paleoAP3 motif. This results in the complete replacement of the paleoAP3 sequence with a novel coding region derived from the 3' UTR and a second indel region. Similar to this case, a frameshift is observed in one of the four paleo*AP3 *paralogs of the magnoliid dicot *Drimys*, which is a recently polyploid genus [51]. The nucleotide identity among these paralogs ranges from 84–93% and three of the four paralogs have canonical paleoAP3 motifs. The fourth, *DrwAP3-1*, diverges in sequence in the second half of the motif, corresponding with an eight nucleotide deletion of this region. It has been argued that compensating mechanisms such as the presence of closely related paralogs or splicing variants can enable frameshift mutations to persist and eventually lead to functional divergence [22,49]. This model is consistent with the current observations for *Paeonia *and *Drimys*, as well as for the ancient eu*AP3*/*TM6 *duplication. The frameshifts detected in *Solanum*, *Paeonia *and *Drimys *may also indicate that this type of event occurs with relative frequency. Although sequence remodeling events such as those in *PesTM6-2 *and *DrwAP3-1 *may very well be lost over a short evolutionary timescale, it only takes one successful event to found a divergent paralogous lineage such as eu*AP3*.

### Molecular 'hopeful monsters'

The term 'hopeful monster' was coined by Goldschmidt [52] to describe new species that arise abruptly by macromutation. Very rarely, he argued, such profound mutations could be beneficial and allow the organism to rapidly adapt to a new mode of life. On the molecular level, the impact of a frameshift mutation on protein sequence is similarly drastic – replacing most, if not all, of the ancestral amino acids with new residues. It seems very likely that the vast majority of such mutations will not be retained, but the eu*AP3*/*TM6 *example, as well as others [22,49], demonstrates that there are isolated cases in which frameshifts have become conserved. Although this phenomenon would seem to be so unlikely as to be vanishingly rare, the role of gene duplication in this process means that it is essentially a matter of numbers, particularly in plants. It has been suggested that plants are especially subject to frequent gene duplications [53], due to everything from genome-scale events to single locus tandem duplications. In particular, loci involved in transcriptional regulation and signal transduction appear to be preferentially retained [54,55]. Phylogenetic analyses of multiple gene families bear out this impression, displaying evidence of duplications at every phylogenetic level (e.g., [27,56-58]). The lower eudicots appear to be a particularly active period for MADS box gene duplication (reviewed [23,59]), leading to the suggestion that at least one genome duplication occurred during this period [60]. Given what may be a relatively high rate of paralog generation, even very rare events such as the appearance of an adaptive frameshift mutation will occur at low frequency. Once such a frameshifted allele appears, it will be subject to the usual microevolutionary forces and may be fixed due to selection or neutral processes. Along these lines, it has been suggested that periods of paralog maintenance due to neutral forces or subfunctionalization may eventually facilitate neofunctionalization [61,62].

Of course, it is only the evolutionarily successful events, or the fairly recent ones, that can be easily detected. Many such molecular 'monsters' may have come and gone over the course of plant evolution. This is not to say that frameshift-based evolution is restricted to plants, since it has also been identified in vertebrates [49]. In these cases, the presence of differentially spliced transcripts is associated with frameshift sequence remodeling. It remains to be seen whether duplication-related frameshift will also be uncovered in animals or if the variable transcript phenomenon will predominate. Other instances of clustered non-synonymous nucleotide changes have been identified [63], which demonstrate that such events can be maintained by selection. These examples may also provide candidates to be re-examined for evidence of frameshift mutation since the failure to recognize a frameshift mutation would result in a nucleotide alignment with the signature of successive non-synonymous substitutions. It is important to note, however, that the 'hopeful monster' analogy only applies to the evolutionary pattern of the protein sequence. At the nucleotide level, the sequence changes are, in fact, quite gradual.

### Implications for the evolution of the *AP3 *lineage and the ABC program

The rapid generation and fixation of the euAP3 motif raises obvious questions regarding its biochemical function and its evolutionary significance. In order to consider these issues, we must first outline our basic knowledge of B gene function in model species. In *Arabidopsis*, AP3 and PI function as obligate heterodimers to promote petal and stamen identity [14,64]. All aspects of their function appear to be interconnected since their heterodimerization through the I and K domains is a requirement for protein stability [65,66], nuclear localization [67], DNA binding [14,68] and the maintenance of gene expression [69,70]. The contribution of the C-terminal motifs to these functions is not well understood. As mentioned previously, it has been demonstrated that the euAP3 motif is required for proper AP3 function and that the paleoAP3 motif is not biochemically equivalent to the euAP3 in *Arabidopsis *[6,32]. The study of Lamb and Irish further determined that the euAP3 motif is capable of conferring AP3-specific function to PI. This result is particularly intriguing since it suggests that dimers between the endogenous PI and chimeric PI_cAP3 _proteins were stabilized when one of the PI proteins possessed a euAP3 motif. Although indirect, this is the best evidence we have to support a role for the euAP3 motif in mediating protein-protein interactions. As to the paleo*AP3 *motif, a study in *Lilium *has argued that this region contributes to the novel homodimerization capacity of the paleo*AP3 *homolog and, further, that the Lilium paleo*AP3 *motif is sufficient to confer homodimerization capability on *AP3 *itself [71]. These findings are highly surprising given that all previous studies have shown that the C domain as a whole plays no role in AP3/PI dimerization [14,16,72]. Additionally, other analyses of both *TM6 *and paleo*AP3 *orthologs have not recovered any evidence of homodimerization [34,36,73,74]. Despite the conflicting nature of this set of results, it remains true that all specific investigations of AP3 motif function have indicated that it plays a role in mediating protein-protein interactions.

Following from this statement, it is natural to now consider the known interaction partners of AP3. The current model of ABCE gene function holds that AP3/PI dimers form higher order complexes with other type II MADS box proteins from the A, C and E classes. In *Arabidopsis*, these genes are represented by *APETALA1 *(*AP1*) in the A class, *AGAMOUS *(*AG*) in the C class and the *SEPALLATA1*-*4 *loci in the E class (reviewed [75]). Therefore, in petals AP3/PI would interact with AP1/SEP dimers and in the stamens, with AG/SEP dimers [76]. This model is assumed to essentially hold for all other core eudicots, with supporting evidence in *Antirrhinum *and *Petunia *[16,77-80]. Unfortunately, the broader findings concerning the functions of C-terminal motifs within the context of these higher order complexes tend to be somewhat contradictory. On the one hand, complete deletion of the motifs does not generally affect complex formation in yeast three- or four-hybrid analyses [16,19] but, on the other hand, a separate yeast three-hybrid study recovered mutations in the C-terminal PI motif that did affect interactions with SEP proteins [17]. Similarly, the ability of *PI*_cAP3 _to rescue *AP3 *function may suggest a role for the euAP3 motif in higher order interactions [6]. Since the C-terminus is not required for AP3/PI dimerization [14], the apparent stabilization of the PI/PI_cAP3 _dimer is unlikely to be due to a direct interaction between the euAP3 motif and PI. It is more probable that the presence of the euAP3 motif allows the weakly associated dimer to interact with other proteins, thereby stabilizing the whole complex. One explanation for this diverse set of results is that there are other proteins participating in complex formation *in planta *that are not represented in the yeast experiments and it is these co-factors that are the targets of C-terminal motif interactions. Alternatively, it may simply be that the yeast system is not always sensitive enough to detect alterations in interaction strength that are significant *in vivo*.

Given that our current understanding of C-terminal motif functions is confusing at best, it is also useful to consider the evolutionary histories of the loci thought to interact with AP3. In the case of *PI*, there is currently no clear evidence for a coincident gene duplication. Moreover, although there are sequence synapomorphies for core eudicot *PI *homologs, none of these map to the C-terminus and the MIK-associated residues do not represent obvious candidates for co-evolutionary changes (Kramer and Hu, unpublished data; [28]). Interestingly, the *AG *and *SEP1*/*4 *lineages both duplicated close to the base of the core eudicots [81,82]. However, AG has been shown to be unable to interact with AP3/PI on its own [19] and neither *AG *nor *SEP1 *underwent any major sequence remodeling in association with their basal eudicot duplications [81,82]. In contrast, the gene lineage containing *AP1 *is of particular interest given that it exhibits an evolutionary pattern which closely parallels that of *AP3 *[48]. Specifically, this lineage duplicated close to the base of the core eudicots to produce the paralogous eu*AP1 *and eu*FUL *lineages. Similar to eu*AP3*, the eu*AP1 *genes are divergent in sequence relative to both eu*FUL *and the ancestral *FUL*-like lineage. Perhaps most surprising is that the remodeling of the eu*AP1 *C-terminus also involved a frameshift mutation, although the exact extent of this phenomenon remains unclear [22,48]. In the case of eu*AP1*, the single ancestral FUL-like motif was lost and two new conserved motifs evolved: one being involved in transcriptional activation (termed the euAP1 motif) and the other a site of post-translational farnesylation [18,20]. No clear data exist, however, regarding the function of the ancestral FUL-like motif or to suggest that the eu*AP1 *motifs play a role in higher order complex formation.

Although it has been proposed that the appearance of the euAP3 and euAP1 motifs may have been a co-evolutionary phenomenon [22], there are at least two variations on this theme that could fit the data. These two hypotheses yield sets of opposing and, most importantly, testable predictions. One possibility is that the new motifs promote interaction with each other in a manner that their ancestors did not. This theory is consistent with the idea that eu*AP1 *and eu*AP3 *acquired their common role in petal identity at the base of the core eudicots [6,22]. Supporting evidence includes the fact that AP1 orthologs can interact with AP3/PI heterodimers on their own, although this does not appear to be dependent on their C-terminal motifs [16,19]. Also, as opposed to the equivocal situation with eu*AP3 *homologs [41], significant data exist to suggest that the role of eu*AP1 *in petal identity is specific to the core eudicots [35,48]. A second scenario is that it was the ancestral FUL-like and paleoAP3 motifs that directly interacted and that, following the gene duplications, the loss of one of these motifs released the other from selection and allowed it to diverge to new function. This theory is more consistent with the lack of data indicating a protein interaction function for the eu*AP1 *motifs. It is interesting to note that the FUL-like motif is strongly similar to the C-terminal motif of the *SEP *lineages [48,81], which are found within the same subfamily as *AP1*/*FUL *[8]. It may be that the loss of the FUL-like motif in eu*AP1 *could be compensated by its conservation in the SEP proteins, which are thought to participate in the same complex. In terms of testable hypotheses, analyses of protein interactions among pre-duplication taxa could help to distinguish between the two models. On the whole, we are left with an intense sense of coincidence – that the *AP3 *and *AP1/FUL *lineages both duplicated and experienced C-terminal frameshift mutation in the same approximate phylogenetic vicinity. Understanding the full significance of this coincidence awaits the definitive establishment of the functions of the C-terminal motifs.

## Conclusion

Phylogenetic analysis of an expanded set of *AP3 *homolog sequences indicates that the eu*AP3*/*TM6 *duplication event occurred very close to the base of the core eudicots in association with the Trochodendraceae and Buxaceae lineages. The current dataset also reveals that the transition from the ancestral paleoAP3 motif to the derived euAP3 motif was primarily mediated by a single nucleotide deletion. The new motif appears to have become conserved with relatively little additional change, a somewhat extraordinary finding highlighting the potential for 'punctuated equilibrium' [83] to act at the molecular level as well as the morphological. It seems likely that the existence of a conserved second paralog facilitated the maintenance of the frameshift mutation. This finding fits with original models of gene duplication as a major source for genetic and biochemical diversification [84]. Current evidence regarding the biochemical functions of these C-terminal motifs is largely indirect and often contradictory, underscoring the importance of targeting these regions for further analysis.

## Methods

### Characterization of *APETALA3 *homologs

Homologs of *AP3 *were cloned from select taxa (see Fig. 1) using reverse transcriptase polymerase chain reaction (RT-PCR) on floral RNA following the protocol described by Stellari et al. [27] and Kramer et al. [28]. 5' rapid amplification of cDNA ends (RACE) was performed on *TroAP3 *using 5' RACE system (Invitrogen^™ ^Life Technologies, Carlsbad CA). Reverse primers are as follows: for the first round of PCR, TroAP3-KR1 5' CTTTTTCCTGTCCGTCTCAGTCTG, and for the second round, TroAP3-KR2 5' TCCACCCGTCCTTCGCCCAATTTC. Sequences have been deposited in GenBank under accession numbers DQ453773-DQ453775 and DQ479353-DQ479368 (see Additional file 1).

### Phylogenetic analyses

In addition to the 20 new loci obtained in the current study, 61 other core eudicot, basal eudicot, magnoliid, monocot and ANITA grade *AP3 *homologs were identified based on previously published analyses and BLAST searches [85] (see Additional file 1 for references and accession numbers). In cases where GenBank contained nearly identical sequences from the same taxon, only one representative sequence was included. Full-length nucleotide alignments of the loci were initially compiled using ClustalW. ClustalW multiple alignment parameters were gap penalty 8 and gap extension penalty 2, transitions weighted for the nucleotide alignment. The alignments were then refined by hand using MacClade 4.06 [86]. The hypothesized single nucleotide deletion in the C-terminus of eu*AP3 *lineage members was incorporated into the alignment (see Additional file 3 for complete alignment in NEXUS format).

Maximum likelihood (ML) phylogenetic analyses were performed using PAUP* [87]. We used Modeltest [88] with the standard Akaike Information Criterion (AIC) to determine the simplest and most appropriate evolutionary model for our dataset. The models selected were a general time-reversible model (GTR) with a proportion of invariable sites (I) and a gamma approximation to the rate of variation among sites (Γ). The ML analysis used a single heuristic search with 100 random addition replicates, TBR branch swapping, MULPARS, and the steepest descent options. Branch support was estimated by performing 100 replicates of nonparametric bootstrapping using the same parameters as the original analysis. We also performed maximum parsimony (MP) analysis on the dataset using a heuristic tree search with 1000 random addition sequence replicates and TBR branch swapping. Support was estimated by performing 1000 bootstrap support replicates each with 10 random sequence addition replicates. The MP phylogeny is not shown (see text).

### Analysis of nucleotide diversity and ancestral character state reconstructions

The program DnaSP [89] was used to determine the position-by-position nucleotide diversity of two small alignments derived from the full-length nucleotide dataset. The first alignment contains the C-terminal paleoAP3 motif-encoding region of loci from the *TM6 *lineage and the paleo*AP3 *lineage of basal eudicots. All indels were removed from the DnaSP alignment (see Additional file 4). The second alignment contains the C-terminal euAP3 motif-encoding region of loci from the eu*AP3 *lineage (all core eudicots). All indels were removed from the DnaSP alignment except for the single nucleotide deletion that produced the euAP3 motif (see Additional file 5). The DNA Polymorphism function was used to determine the nucleotide diversity (π, [90]) for each position in the two alignments.

Ancestral nucleotide character state reconstructions were performed using both MP and ML methods. For these analyses, we used the complete nucleotide alignment and the ML phylogeny. MP reconstructions were performed using the accelerated transitions (ACCTRAN) and delayed transitions (DELTRAN) options as they are implemented in MacClade 4.0 [86]. ML reconstructions were performed using the approach of Yang et al. [91] that is implemented in PAML [92]. As has been found in other cases where changes are relatively rare ([93] and references therein), the MP and ML reconstructions were identical. Given the fact that the relevant nodes have poor support, we also performed ancestral character state reconstructions with alternative topologies. Specifically, we tested a phylogeny where the *Pachysandra *loci are placed before the eu*AP3*/*TM6 *duplication (see Fig. 5B). In addition, we rearranged the eu*AP3 *and *TM6 *clade members such that their relationships were consistent with published core eudicot relationships. For this set of analyses, we tried two alternative topologies, one consistent with Soltis et al. 2003 [42] and the other, with Kim et al. [43].

## Authors' contributions

EK characterized *AP3 *homologs from *Ilex*, *Kalanchoe*, *Saxifraga*, *Corylopsis*, *Pachysandra*, *Phytolacca*, *Paeonia *and *Vitis*; conducted the phylogenetic analyses and ancestral state reconstructions; and drafted the manuscript. HJS and JMH characterized *AP3 *homologs from *Loranthus *and *Trochodendron*; and helped draft the manuscript. CCW characterized the *AP3 *homolog from *Nelumbo *in the laboratory of JMH. All authors read and approved of the final manuscript.

## Supplementary Material

Additional file 1 **Table with Locus information **Taxa of origin, GenBank accession numbers and reference information for all loci included in the alignment (sorted alphabetically by taxon). Click here for file

Additional file 2 **Alignment of C-terminal regions of predicted proteins of paleo*AP3*, *TM6 *and eu*AP3 *representatives. **Phylogenetic affinities of the taxa are indicated by the bars on the left (BE = Basal Eudicot; based on [42, 95]. The PI Motif-derived region is boxed in green; paleoAP3 motifs, in blue; and euAP3 motifs, in purple. Residues showing chemical conservation with the consensus for each of these regions [28] are shaded in grey. Red arrows at the right indicate the loci that appear to have experienced independent frameshift mutations. Click here for file

Additional file 3 ***APETALA3 *nucleotide alignment **NEXUS format file of complete *APETALA3 *nucleotide alignment used in current phylogenetic analyses. Click here for file

Additional file 6 **Comparison of position-by-position nucleotide diversity values for paleoAP3 and euAP3 motif containing loci. **Complete dataset of nucleotide diversity values of paleoAP3 and euAP3 containing loci. Region spans the entire C-terminal motif. The yellow bars indicate the values for a dataset including all *TM6 *lineage members and basal eudicot paleo*AP3 *loci. The codon positions of each nucleotide are indicated by vertical hash marks and the corresponding amino acids are shown immediately below the chart. Note that the last four nucleotides in the paleoAP3 alignment are 3' UTR. The blue bars indicate the values for a dataset including all eu*AP3 *lineage members. The codon positions of each nucleotide are indicated by vertical hash marks and the corresponding amino acid are shown at the bottom. The position of the euAP3 frameshift is represented by a dash mark. n/a = not applicable. Click here for file

Additional file 4 **PaleoAP3 alignment for nucleotide diversity calculation **Alignment of paleoAP3 encoding regions of *Pachysandra *loci, *TM6 *orthologs and basal eudicot paleo*AP3 *representatives. Indels were removed from the alignment. Click here for file

Additional file 5 **EuAP3 alignment for nucleotide diversity calculation **Alignment of euAP3 motif encoding regions of eu*AP3 *lineage members. All indels were removed except for the single nucleotide deletion corresponding to the euAP3 motif frameshift. Click here for file
