# Supplementary material for: A simplified explanation for the frameshift mutation that created a novel C-terminal motif in the APETALA3 gene lineage
Source: BMC Evol Biol. 2006 Mar 24;6:30. doi: 10.1186/1471-2148-6-30 (PMC1513400; doi:10.1186/1471-2148-6-30)
Supplement: Additional file 1 — Table with Locus information Taxa of origin, GenBank accession numbers and reference information for all loci included in the alignment (sorted alphabetically by taxon). [file 1471-2148-6-30-S1.doc]

# Additional File 1

Table 1: Sampling and GenBank Accession Information

| **Locus** | **Taxon** | **Voucher #*** | **Acc. #** | **Ref.** |
| --- | --- | --- | --- | --- |
| *AkqAP3-1* | *Akebia quinata* |  | AY162835 | [22] |
| *AkqAP3-2* | *Akebia quinata* |  | AY162838 | [22] |
| DefA | Antirrhinum majus |  | X62810 | [1] |
| *AqAP3-1* | *Aquilegia alpina* |  | AY162849 | [22] |
| *AqAP3-2* | *Aquilegia alpina* |  | AY162850 | [22] |
| *AqAP3-3* | *Aquilegia alpina* |  | AY162851 | [22] |
| *AP3* | *Arabidopsis thaliana* |  | M86357 | [11] |
| *AsAP3* | Argyroxiphium sandwicense |  | AAD51197 | [19] |
| *AreAP3* | *Aristolochia eriantha* |  | AY436714 | [23] |
| *BgAP3-1* | *Berberis gilgiana* |  | AY162857 | [22] |
| *BgAP3-2* | *Berberis gilgiana* |  | AY162858 | [22] |
| *Boi1AP3* | *Brassica oleracea* |  | U67453 | [12] |
| *CfAP3-1* | *Calycanthus floridus* |  | AF230699 | [6] |
| *CfAP3-2* | *Calycanthus floridus* |  | AF230700 | [6] |
| *CsAP3* | *Chloranthus spicatus* |  | AF230701 | [6] |
| *CDM115* | *Chrysanthemum x morifolium* |  | AY173060 | Unpub. |
| *CDM19* | *Chrysanthemum x morifolium* |  | AY173064 | Unpub. |
| *CopAP3* | *Corylopsis pauciflora* | AA 894-81 | DQ479353 | This study |
| *CopTM6-1* | *Corylopsis pauciflora* | AA 894-81 | DQ479354 | This study |
| *CopTM6-2* | *Corylopsis pauciflora* | AA 894-81 | DQ479355 | This study |
| *DcMADS3* | *Daucus carota* |  | AJ271149 | [8] |
| *CMB2* | Dianthus caryophyllus |  | L40405 | [16] |
| *DeAP3* | *Dicentra eximia* |  | AF052875 | [2] |
| *DrwAP3-1* | *Drimys winteri* |  | AY436722 | [23] |
| *DrwAP3-4* | *Drimys winteri* |  | AY436725 | [23] |
| *GDEF1* | *Gerbera hybrida* |  | AJ009724 | [7] |
| *GDEF2* | *Gerbera hybrida* |  | AJ009725 | [7] |
| *GtAP3-1* | Gunnera tinctoria |  | AY337753 | [14] |
| *GtAP3-3* | *Gunnera tinctoria* |  | AY337755 | [14] |
| *HemMADS1* | *Hemerocallis* hybrid cultivar |  | AF209729 | Unpub. |
| *HPDEF1* | *Hieracium piloselloides* |  | AF180364 | Unpub. |
| *HmAP3* | *Hydragea macrophylla* |  | AF230702 | [6] |
| *HmTM6* | *Hydrangea macrophylla* |  | AF230703 | [6] |
| *IxaAP3* | *Ilex aquifolium* | AA 11-91 | DQ479356 | This study |
| *IxaTM6* | *Ilex aquifolium* | AA 11-91 | DQ479357 | This study |
| *JrAP3* | Juglans regia |  | AJ313089 | Unpub. |
| *KbAP3-1* | Kalanchoe blossfeldiana | EMK221 | DQ479359 | This study |
| *KbAP3-2* | Kalanchoe blossfeldiana | EMK221 | DQ479358 | This study |
| *LodTM6* | *Loranthus delavayi* | CCWu 0033 | DQ453773 | This study |
| *MpMADS7* | Magnolia praecocissima |  | AB050649 | Unpub. |
| *MADS13* | *Malus domestica* |  | CAC80856 | Unpub. |
| *MdTM6* | *Malus domestica* |  | AB081093 | [21] |
| *NMH7* | *Medicago sativa* |  | L41727 | [13] |
| *MdAP3-1* | *Meliosma dilleniifolia* |  | AY436709 | [23] |
| **Locus** | **Taxon** | **Voucher #*** | **Acc. #** | **Ref.** |
| *MdAP3-2* | *Meliosma dilleniifolia* |  | AY436710 | [23] |
| *MdAP3-3* | *Meliosma dilleniifolia* |  | AY436711 | [23] |
| *NnAP3* | *Nelumbo nucifera* | CCWu 0016 | DQ453775 | This study |
| *NTDEF* | *Nicotiana tobaccum* |  | X96428 | [5] |
| *NymAP3* | *Nymphaea* sp. |  | AY436740 | [23] |
| *PpAP3-1* | *Pachysandra procumbens* | AA 444-43 | DQ479360 | This study |
| *PpAP3-2* | *Pachysandra procumbens* | AA 444-43 | DQ479361 | This study |
| *PpAP3-3* | *Pachysandra procumbens* | AA 444-43 | DQ479362 | This study |
| *PtAP3-1* | *Pachysandra terminalis* |  | AF052870 | [2] |
| *PtAP3-2* | *Pachysandra terminalis* |  | AF052871 | [2] |
| *PesTM6-1* | Paeonia suffruticosa | AA 937-70 | DQ479363 | This study |
| *PesTM6-2* | *Paeonia suffruticosa* | AA 937-70 | DQ479364 | This study |
| *PhTM6* | *Petunia hybrida* |  | AF230704 | [2] |
| *PMADS1* | *Petunia hybrida* |  | X69946 | [3] |
| *PhaTM6-1* | *Phytolacca americana* | EMK220 | DQ479365 | This study |
| *PhaTM6-2* | *Phytolacca americana* | EMK220 | DQ479366 | This study |
| *PloAP3-1* | *Platanus occidentalis* |  | AY162881 | [22] |
| *PloAP3-2* | *Platanus occidentalis* |  | AY162882 | [22] |
| *PTD* | *Populus balsamifera* |  | AF057708 | [18] |
| *RsAP3-1* | Ribes sanguineum |  | AY337758 | [14] |
| *MASAKOB3* | Rosa rugosa |  | AB055966 | [20] |
| *RAD1* | *Rumex acetosa* |  | U28482 | [10] |
| *RAD2* | *Rumex acetosa* |  | U28484 | [10] |
| *ScAP3* | *Sanguinaria canadensis* |  | AF130868 | [2] |
| *SrhAP3* | *Saruma henryii* |  | AY436715 | [23] |
| *SxcAP3* | *Saxifraga careyana* | EMK202 | DQ479367 | This study |
| *SxcTM6* | *Saxifraga careyana* | EMK202 | DQ479368 | This study |
| *SlAP3A* | *Silene latifolia* |  | AB090863 | [17] |
| *SlAP3Y* | *Silene latifolia* |  | AB090864 | [17] |
| *SLM3* | *Silene latifolia* |  | X80490 | [9] |
| *LeAP3* | *Solanum lycopersicon* |  | AF052868 | [2] |
| *TM6* | *Solanum lycopersicon* |  | X60759 | [15] |
| *StDef* | *Solanum tuberosum* |  | X67508 | [4] |
| *SvAP3* | *Syringa vulgaris* |  | AF052869 | [2] |
| *TcAP3* | Tacca chantieri |  | AF230706 | [6] |
| *TroAP3* | Trochodendron aralioides | JMHu 1356 | DQ453774 | This study |

* Voucher information is only provided for taxa in the current study.

# References Cited

1. Schwarz-Sommer Z, Hue I, Huijser P, Flor PJ, Hansen R, Tetens F, Lonnig W-E, Saedler H, Sommer H: **Characterization of the *Antirrhinum* floral homeotic MADS-box gene *deficiens*: evidence for DNA binding and autoregulation of its persistent expression throughout flower development.** *EMBO J* 1992, **11**(1):251-263.

2. Kramer EM, Dorit RL, Irish VF: **Molecular evolution of genes controlling petal and stamen development: Duplication and divergence within the *APETALA3* and *PISTILLATA* MADS-box gene lineages.** *Genetics* 1998, **149**:765-783.

3. Kush A, Brunelle A, Shevell D, Chua N-H: **The cDNA sequence of two MADS box proteins in petunia.** *Plant Physiology* 1993, **102**:1051-1052.

4. Garcia-Maroto F, Salamini F, Rohde W: **Molecular cloning and expression patterns of the *Deficiens* homologous gene *St-Deficiens* from *Solanum tuberosum***. *Plant J* 1993, **4**:771-780.

5. Davies B, Di Rosa A, Eneva T, Saedler H, Sommer H: **Alteration of tobacco floral organ identity by expression of combinations of *Antirrhinum* MADS-box genes.** *Plant Journal* 1996, **10**(4):663-677.

6. Kramer EM, Irish VF: **Evolution of the petal and stamen developmental programs: Evidence from comparative studies of the lower eudicots and basal angiosperms**. *Int J Plant Sci* 2000, **161**(6 Suppl.):S29-S40.

7. Yu D, Kotilainen M, Pollanen E, Mehto M, Elomaa P, Helariutta Y, Albert A, Teeri T: **Organ identity genes and modified patterns of flower development in *Gerbera hybrida* (Asteraceae).** *Plant Journal* 1999, **17**:51-62.

8. Linke B, Nothnagel T, Borner T: **Flower development in carrot CMS plants: mitochondria affect the expression of MADS box genes homologous to *GLOBOSA* and *DEFICIENS*.** *Plant J* 2003, **34**:27-37.

9. Hardenack S, Ye D, Saedler H, Grant S: **Comparison of MADS box gene expression in developing male and female flowers of the dioecious plant white campion.** *Plant Cell* 1994, **6**:1775-1787.

10. Ainsworth C, Crossley S, Buchanan-Wollaston V, Thangavelu M, Parker J: **Male and female flowers of the dioecious plant sorrel show different patterns of MADS box gene expression.** *Plant Cell* 1995, **7**:1583-1598.

11. Jack T, Brockman LL, Meyerowitz EM: **The homeotic gene APETALA3 of Arabidopsis thaliana encodes a MADS box and is expressed in petals and stamens.** *Cell* 1992, **68**:683-697.

12. Carr S, Irish V: **Floral homeotic gene expression defines developmental arrest stages in *Brassica oleracea* L. vars. *botrytis* and *italica*.** *Planta* 1997, **201**(2):179-188.

13. Heard J, Dunn K: **Symbiotic induction of a MADS-box gene during development of alfalfa root nodules.** *Proc Natl Acad Sci* 1995, **92**:5273-5277.

14. Kim S, Yoo M, Albert VA, Farris JS, Soltis PS, Soltis DE: **Phylogeny and diversification of B-function genes in angiosperms: Evolutionary and functional implications of a 260-million year old duplication.** *Am J Bot* 2004, **91**(12):2102-2118.

15. Pnueli L, Abu-Abeid M, Zamir D, Nacken W, Schwarz-Sommer Z, Lifschitz E: **The MADS box gene family in tomato: temporal expression during floral development, conserved secondary structures and homology with homeotic genes from *Antirrhinum* and *Arabidopsis*.** *The Plant Journal* 1991, **1**(2):255-266.

16. Baudinette SC, Stevenson TW, Savin KW: **Isolation and characterisation of the carnation floral-specific MADS box gene, CMB2.** *Plant Sci* 2000, **155**(2):123-131.

17. Matsunaga S, Isono E, Kejnovsky E, Vyskot B, Dolezel J, Kawano S, Charlesworth D: **Duplicative transfer of a MADS box gene to a plant Y chromosome**. *Mol Biol Evol* 2003, **20**(7):1062-1069.

18. Sheppard LA, Brunner AM, Krutovskii KV, Rottmann WH, Skinner JS, Vollmer SS, Strauss SH: **A *DEFICIENS* homolog from the dioecious tree Black Cottonwood is expressed in female and male floral meristems of the two-whorled, unisexual flowers**. *Plant Physiology* 2000, **124**:627-639.

19. Barrier M, Baldwin BG, Robichaux RH, Purugganan MD: **Interspecific hybrid ancestry of a plant adaptive radiation: Allopolyploidy of the Hawaiian Silversword alliance (Asteraceae) inferred from floral homeotic gene duplications.** *Molecular Biology and Evolution* 1999, **16**:1105-1113.

20. Kitahara K, Hirai S, Fukui H, Matsumoto S: **Rose MADS-box genes 'MASAKO BP and B3' homologous to class B floral identity genes.** *Plant Sci* 2001, **161**:549-557.

21. Kitahara K, Ohtsubo T, Soejima J, Matsumoto S: **Cloning and characterization of apple class MADS-box genes including a novel AP3 homologue MdTM6**. *Journal of the Japanese Society for Horticultural Science* 2004, **73**(3):208-215.

22. Kramer EM, Di Stilio VS, Schluter P: **Complex patterns of gene duplication in the APETALA3 and PISTILLATA lineages of the Ranunculaceae.** *IJPS* 2003, **164**(1):1-11.

23. Stellari GM, Jaramillo MA, Kramer EM: **Evolution of the *APETALA3* and *PISTILLATA* lineages of MADS-box containing genes in basal angiosperms.** *Mol Biol Evol* 2004, **21**(3):506-519.
